# Multi-temporal assessment of a wildfire chronosequence by remote sensing

**DOI:** 10.1016/j.mex.2024.103011

**Published:** 2024-10-18

**Authors:** F. Nájera De Ferrari, E. Duarte, C. Smith-Ramírez, A. Rendon-Funes, V. Sepúlveda Gonzalez, N. Sepúlveda Gonzalez, M.F. Levio, R. Rubilar, A. Stehr, C. Merino, I. Jofré, C. Rojas, F. Aburto, Y. Kuzyakov, E. Filimonenko, J. Dörner, P. Pereira, F. Matus

**Affiliations:** a Laboratorio de Conservación y Dinámica de Suelos Volcánicos, Departamento de Ciencias Químicas y Recursos Naturales, Facultad de Ingeniería y Ciencias, Universidad de La Frontera, Chile; b Laboratorio de Geomicrobiología, Facultad de Ingeniería y Ciencias, Departamento de Ciencias Químicas y Recursos Naturales, Universidad de La Frontera, Chile; c Departamento de Ciencias Biológicas y Biodiversidad, Universidad de Los Lagos, Chile; d Instituto de Ecología y Biodiversidad-Chile (IEB), Chile; e Instituto de Conservación, Biodiversidad y Territorio, Facultad de Ciencias Forestales y Recursos Naturales, Universidad Austral de Chile, Chile; f Facultad de Ciencias Forestales, Universidad de Concepción, Chile; g Departamento de Ingeniería Civil, Facultad de Ingeniería, Universidad de Concepción, Chile; h Scientific and Biotechnological Resources Nucleus, Universidad de La Frontera (Bioren, UFRO), Chile; i Instituto de Ciencias Agroalimentarias, Animales y Ambientales de la Universidad de O'Higgins, Chile; j Department of Soil and Crop Sciences, AgriLife Research, Texas A&M University, USA; k Departamento de Planificación Territorial y Sistemas Urbanos, Facultad de Ciencias Ambientales, Universidad de Concepción, Chile; l Department of Soil Science of Temperate Ecosystems, Department of Agricultural Soil Science, University of Gottingen, 37077 Gottingen, Germany; m Peoples Friendship University of Russia (RUDN University), 117198 Moscow, Russia; n University of Tyumen, Volodarskogo str., 6, Tyumen 625003, Russia; o Instituto de Ingeniería Agraria y Suelos, Universidad Austral de Chile, Chile. Centro de Investigación en Suelos Volcánicos, Chile; p Environmental Management Laboratory, Mykolas Romeris University, Vilnius, Lithuania

**Keywords:** Forest fires burn severity, Temporal assessment, Remote sensing, Assessment of wildfire chronosequence

## Abstract

The study aimed to develop a methodological framework to identify forest ecosystems affected by wildfires and evaluate their recovery chronologically. To do this remote sensing analysis, sites with burn scars were selected based on various criteria (fire severity, affected area, vegetation and soil type, slope, aspect, and one-time occurrence of wildfire in the last 23 years). Spectral vegetation indices (VIs) from satellite imagery were used to estimate burn severity and vegetation cover changes. Images of surface reflectance were obtained from the collection of Landsat 5 ETM, Landsat 7 ETM+, and Landsat 8 OLI/TIRS, available and processed on the Google Earth Engine Platform (GEE). Indices VIs (i) the normalized difference vegetation index (NDVI), (ii) the normalized burn ratio (NBR), and (iii) the differenced normalized burn ratio (dNBR) were calculated to classify burn severity. The one-time occurrence selection was performed using the LandTrendr algorithm to monitor changes in land cover and burned areas. To validate the selection, the chosen sites within the chronosequence were clustered on 4 seasons of soil properties and litter accumulation recovery. Our result can guide methodological comparisons and forest management practices on large surfaces by comparing parches of different time-affected ecosystems. Validation sites of the cluster chronosequence shows consistent recovery of soil properties as soil carbon, bulk density and litter accumulation through the studied years

• The study developed a framework to identify wildfire-affected forest ecosystems and evaluate their recovery using remote sensing and local data.
• Vegetation indices (NDVI, NBR, dNBR) from Landsat satellite imagery processed on the Google Earth Engine were used to assess burn severity and vegetation changes over time.
• Selected sites were validated using the LandTrendr algorithm and monitored for seasonal changes in soil properties and litter accumulation.

## Graphical abstract

The selection sites affected by wildfire under a chronosequence was firths identified by the burn scars and fire severity determination, followed by the temporal parameters were evaluated by plant-grown stages assessment and all sites were validated in-situ by soils parameters.

**Figure:**
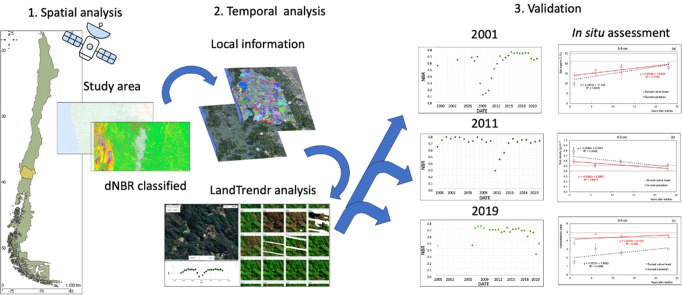

Specifications table

**Table:** 

Subject area:	Environmental Science
More specific subject area:	Burn severity assessment and recovery evaluation of forest areas.
Name of your method:	Assessment of wildfire chronosequence
Name and reference of original method:	Keeley, J. E. (2009). Fire intensity, fire severity and burn severity: A brief review and suggested usage. International Journal of Wildland Fire, 18(1), 116–126. doi:10.1071/WF07049 Kennedy, R.E., Yang, Z. & Cohen, W.B. (2010). Detecting trends in forest disturbance and recovery using yearly Landsat time series: 1. LandTrendr - Temporal segmentation algorithms. Remote Sensing of Environment, 114, 2897–2910. doi:10.1016/j.rse.2010.07.008 Kennedy, R.E., Yang, Z., Gorelick, N., Braaten, J., Cavalcante, L., Cohen, W.B., Healey, S. (2018). Implementation of the LandTrendr Algorithm on Google Earth Engine. Remote Sens. 2018, 10, 691. doi:10.3390/rs10050691
Resource availability:	Computer AMD RYZEN(R) 5 5500 U with Radeon Graphics 2.10 GHz and 16.0 GB Memory; Google earth engine (http://earthengine.google.com); QGIS desktop 3.36.2.; Time series analysis algorithm (LandTrendr) developed by Braaten J (2021) https://github.com/jdbcode/ee-rgb-timeseries?tab=Apache-2.0–1-ov-file; Chilean Geospatial information available free www.geoportal.cl/catalog

## Background

Wildfires are natural phenomena that cause immediate changes in the ecosystems. Wildfires are associated with the loss of lives and infrastructure, greenhouse gas emissions, soil degradation, and the destruction of species, biomass, biodiversity and the water balance [[1], [2], [3]].

Wildfire severity (the impact of wildfires on ecosystems) depends on the ecosystem's intrinsic characteristics, such as soil and forest type, biomass accumulation, and pre-fire land use [4]. After a fire, recovery depends on the severity of the burn, topography, and meteorological and climate conditions. Because of those characteristics, remote sensing analyses are being increasingly used to evaluate wildfires from the beginning of the event to several years after vegetation recovery.

There are increasing studies of spatial analysis of vegetation recovery after fire. Mapping and modelling post-fire vegetation recuperation are crucial in spatial forest ecology. Monitoring post-fire vegetation recovery (PVR) helps to identify ecosystem resilience and landscape dynamics and gives guidelines for forest management purposes [5]. Geospatial assessment, for example, using satellite remote sensing, can increase the insight into the impacts of wildfire on the vegetation cover [[6], [7], [8], [9]]. Spectral vegetation indices (VIs) derived from satellite multispectral imagery are a key part of these methods. These VIs are proxies of forest structure because of the intensity of the radiation of specific bands of the electromagnetic spectrum that vegetation emits or reflects [10]. The Vis can be influenced by the type of forest but determined mainly by the fraction of consumed canopy after fire [[11], [12], [13]]. Monitoring terrestrial natural resources requires a long series of satellite imagery-derived products. Nowadays, there is a challenge in processing large volumes of data. Google Earth Engine (GEE) is a cloud-computing platform tool for analyzing geospatial information, which makes it possible to process large time-series datasets for vegetation monitoring. These databases can be queried and analyzed in the cloud, reducing the requirement for local computing infrastructure [14]. GEE combines >40 years of historical and current satellite images from various sensors (e.g., Landsat 8, Sentinel-2), continuously updated (about 6000 new scenes daily). In Chile, most of the burned forest and plantation areas occur in the Andes and Coastal Cordillera, where the accessibility to combat wildfires is high [15], thus increasing the use of remote sensing analyses. Related works on wildfires are being found to improve the accuracy of the existing data [16] and complement remote sensing analysis with vegetation field-based observational data [17,18].

Wildfires affect soils on their physical and chemical properties, such as organic carbon content, bulk density, soil aggregate stability, hydrophobicity, and pH, even at low burn severity [19]. During and after burning, soil microbial communities and soil minerals, such as smectite and kaolinite/chlorite composition [20]. Meanwhile, management after a wildfire as the vegetation removal increases erosion and overland flow [21,22]. Our proposed methodology reports specific periods, vegetation grown, and soil properties to find and select ecosystems affected by wildfires in areas that are similar to those affected by historical land use and management, with the aim of evaluating the common patterns of forest ecosystem recovery.

## Methodology

The present study was developed as a methodological framework for assessing forests affected by wildfires following a chronosequence of PVR, as it is show on Table 1. Medium-high-severity burned forest sites were selected over 23 years. Remote sensing analysis was performed to identify the burn scar severity [23] and to evaluate the soil and vegetation recovery. The LandTrendr analysis algorithm was used to identify changes in pixel values over time in vegetation recovery [24] in the platform GEE. Local field vegetation survey information was compiled to fulfil several criteria for selecting medium- to high-severity wildfires on forest sites. Each site was validated in situ by soil sampling and vegetational inventory.

**Table 1:** framework of selecting and validating sites affected by wildfire in a chronosequence determined by plant-grown stages.

Steps	Remote sensing analyses	
Burn scars analyses	Goggle Earth Engine	Burn severity Ratio
Selection criteria	QGIS 3.2	Digital elevation model (DEM) for altitude, exposition, and slope analyses. Land use survey- forest sub-use native forest and plantation, soil information.
Fire chronosequence analyses	Goggle Earth Engine	Time series analysis algorithm (LandTrendr) developed by Braaten J (2021)
	Validation	
Field analyses	Fieldwork	Soil sampling and vegetation cover evaluation

### Study site description

The study was conducted in the La Araucania region, Chile (38°54′S, 72°40′O) with >100,000 ha of native forest (mainly evergreen species.) and >90,000 ha of plantation (mainly *Pinus radiata Don*) affected by wildfires during the last 22 years [25].^1^ Wildfires have been increasing consistently since 2010 due to rising temperatures and decreasing precipitations[26,27]. Native forest, covering approximately one million hectares, represents 24.5 % of the total area of the La Araucania Region. Only 10 % of this forested area is protected by national parks. Exotic tree plantations represent 14.5 % of the La Araucania region and 38 % of the total productive surface of the country [28]. Since 2000, 39 % of the affected surface has been forest plantation (mainly *Pinus* sp*.)*, and 52 % on other vegetation types such as grasses, native forests, shrublands, and understory. Most of the native forests had dense coverage of secondary growth from historical management before the year 84. Elevation varied between 300 at 1800 m a.s.l. The dominant sites' slope exposures were west and northeast, where the sun hits more directly over the year in the south hemisphere. The average climate in the area indicates a pluviometry of >2000 mm per year, and the maximum mean temperature in summer is 25 °C [29].

### Burn severity assessment

**Fig. 1:**
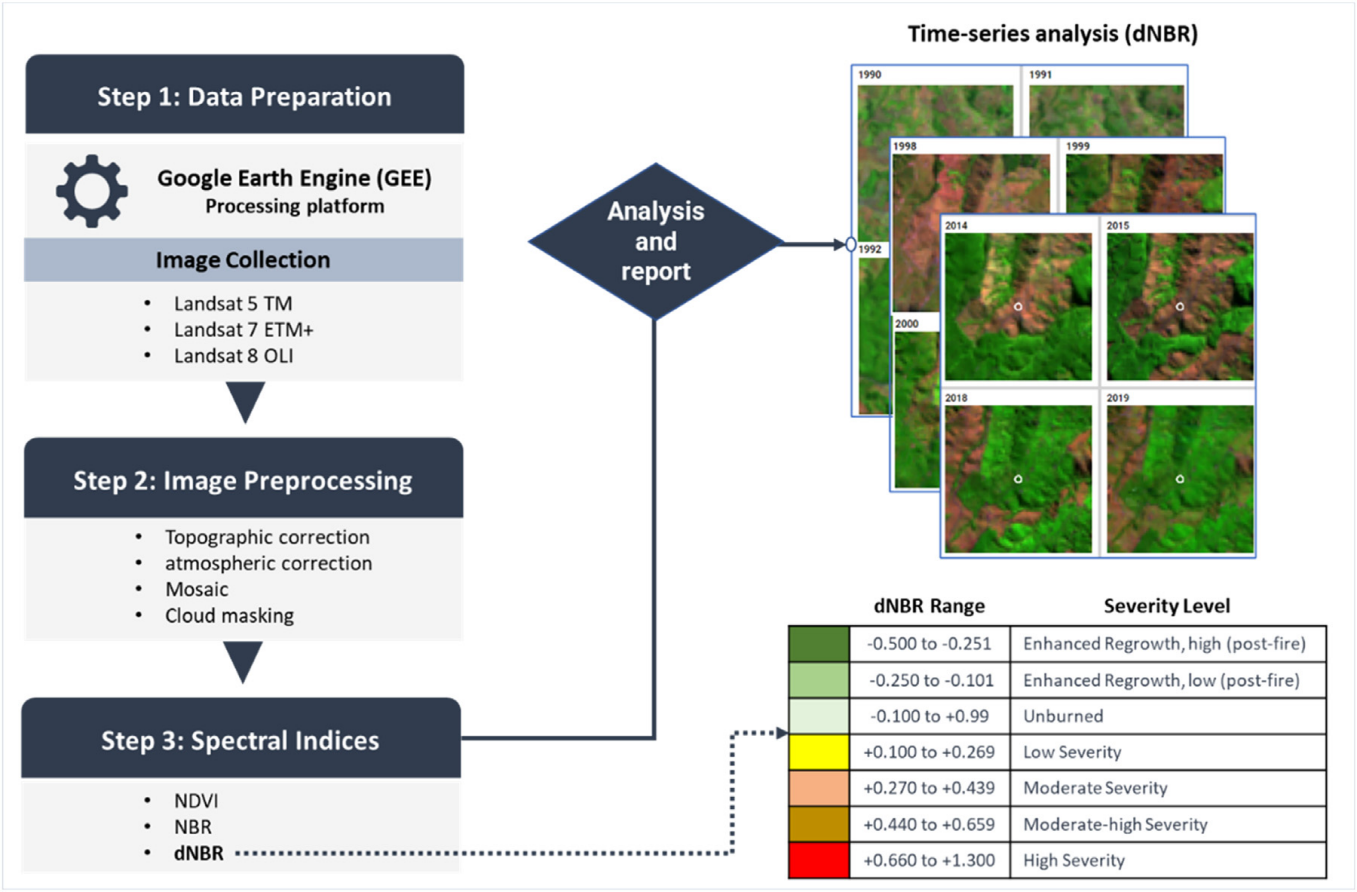
Workflow for image preprocessing and wildfire chronosequence estimation: Here, satellite images were selected to determine the dNBR index to classify burn severity levels according to the United States Geological Survey (USGS) classification. The images were selected and processed on the online platform Google Earth Engine using the methodology proposed by [23].

We used Google Earth Engine (GEE) public catalogue of satellite images to generate a detailed database of fire scars. Landsat satellite images were used since they have brooded years of coverage of the studied area (1985 to the present). Thus, Landsat 5 and 7 images were processed by the Landsat Ecosystem Disturbance Adaptive Processing System (LEDAPS), and Landsat 8 images were processed by the Landsat 8 Surface Reflectance (L8SR) system. Imagery with cloudy pixels and cloud shadows was removed by using the Fmask algorithm, with more than 50 % of shadows and cloud coverage [30]. To obtain a Burn Severity map for our study area, we followed method on [31] and the burn severity levels as per the United States Geological Survey (USGS) classification, we estimated the normalized difference vegetation index (NDVI), Normalized Burn Ratio (NBR), and calculated the differenced NBR (dNBR index). (Fig. 1). The normalized difference vegetation index NDVI is commonly used to estimate the quantity, quality, and development of vegetation [10] and the normalized burn ratio (NRB) is calculated as the ratio between the NBR and short-wave infrared (SWIR) to quantify burning severity. NBR is closely related to the water content in vegetation and soil, characterized by the SWIR band [23]. The difference between pre-and post-fire NBR is used to calculate delta NBR (dNBR or ∆NBR), which can then be used to estimate the burn severity (Table 2). A higher dNBR value indicates high damage, while a lower dNBR suggests low damage. Negative values may indicate vegetation regeneration after a wildfire [32]. previous studies highlighted that indices derived from SWIR perform better than those based on the red and near-infrared bands [[33], [34], [35]]. Assessing the differences between forests grown in areas where recovery rates differ for different ages is particularly important. The dNBR was classified according to the United States Geological Survey (USGS) standard for Burn Severity assessment. Spatial analysis was performed with the available images in Google Earth Engine (GEE) platform.

**Table 2:** Derived indices from remote sensing used to identify and evaluate burned areas.

Vegetation index (VI)	Formula^a^
Normalized difference vegetation index (NDVI)	$NDVI=\frac{\text{NIR}-\text{RED}}{\text{NIR}+\text{RED}}$
Normalized Burn Ratio (NBR)	$NBR=\frac{\text{NIR}-\text{SWIR}}{\text{NIR}+\text{SWIR}}$
differenced Normalized Burn Ratio (dNBR)	$dNBR=NBR_{\left(prefire\right)}-\;NBR_{\left(postfire\right)}$

a NIR: Near InfraRed band; SWIR: Short Wavelength InfraRed band; RED: color red band.

Each year studied, we selected images of pre-fire and post-fire seasons. Fire season in the south hemisphere goes from the spring (October) of the first year to the beginning of fall (March). We filtered images with few clouds (<5 %) for pre-fire image selection between March and June. Post-fire images were also filtered for cloud coverage with medium to high-severity fire scars from October to March next year. In total, we obtained burn severity maps for each year of the study.

### Selection criteria

The burn scar images with the severity index were filtered with local information in the QGIS 3.12 program and were selected to choose field sites for in situ validation. Geographical and survey information from official governmental sources were analyzed to evaluate whether the specific criteria applied for the site selection were fulfilled. Nine selection criteria based on expert knowledge and geographical characteristics were considered (Table 3).

**Table 3:** Selection criteria for a wildfire in a native forest and pine plantation that just burned once in the chronosequence.

Criteria	Description	Selection
1	Native forest formation dominated by evergreen trees *a*nd *P. radiata,* pine plantation.	The botanical composition, the age of the forest, and the surface coverage by the canopy. Pine plantation was selected by the age of the plantation (<10 years) and height of the trees (<4 m).
2	Soil properties	Soils derived from volcanic materials classified as Andosols.
3	Fire description	A medium to high severity was selected. The form of the fire in the and the surrounding fire-affected sites with lower severity classification was analyzed.
4	Sites extension	Selective sites were >2 hectares of area.
5	Elevation	< 2000 m a.s.l to avoid the no vegetation land
6	Slope	5 to 40 %
7	Aspect	North slope, Northeast, Northwest
8	Road distance	< 5 km
9	Fire distances	Distance between fires to avoid sites burned frequently.

The main criteria were: 1) Vegetation type: In this study, the vegetation was selected as the most abundant on areas, to understand shared forest recovery patters, reducing vegetation type interference on the remote sensing analyses [36]. The botanical composition, forest age, and the surface coverage by the canopy were considered. The pine plantation was selected according to the trees' age and height. The data to identify native forests and pine plantations was obtained from the digital land use survey [37]. 2) Soil properties: Chemical, biological and physical properties from recent volcanic ash-derived soil, i.e. Andisols [38], for both native forests and pine plantation stands were regarded. Soil information was obtained from [39]. 3) Burn severity index classification was used to identify wildfires with medium to high-severity patches surrounded by medium to low-severity areas, which identified wildfire shapes and affected areas. 4) Plot size: An area of a minimum of two hectares was established to ensure a minimum area for sampling criteria of a medium to high-severity wildfire, to assess soil properties and vegetation recovery, and to avoid border effects. Topography variables, such as elevation, slope, and aspect, were selected for frame vegetation formation types, fire occurrences as are more frequent on the north aspect of the mountain, and slope to relate soil erosion after a fire (Table 3). Vegetation inventory, soil map and geographical features are shown (Table S1, Map S1, Soil classification, Map S2 slope, Map S3 aspects, Supplementary Materials). The sites that fulfil the selection were evaluated to elaborate the chronosequence assortment of one wildfire event that occurred on 23 years of vegetational growth with the LandTrendr analyses. The initial condition of one-time burning sites was chosen to ensure similar starting points for the recovery of native forests, accumulation of litter under similar species growth, and restoration of soil nutrients over the 23-year chronosequence. The recovery timing after a fire is crucial in determining fire severity to avoid the impact of several fires in one place [40].

After the selection, in the study area, not many highly severe fires that fulfilled the selection criteria were found in each of the studied years. Consequently, we had five seasons for vegetation recovery and four seasons for soil sampling intervals were established. Therefore, the wildfire chronosequence intervals for sampling were determined by considering the recovery stages of pine plantation and native forest vegetation. The ecosystem recovery in this study was determined for the wildfire intervals of 2023–2021, 2017–2019, 2011–2012, 2005–2006, and 2001–2002, equivalent to 0, 6,12, 16 and 23 years after the wildfire event, respectively. The evaluated properties are variables from one season to another, particularly for the first two years after the wildfire [41,42].

### Fire chronosequence analysis

**Fig. 2:**
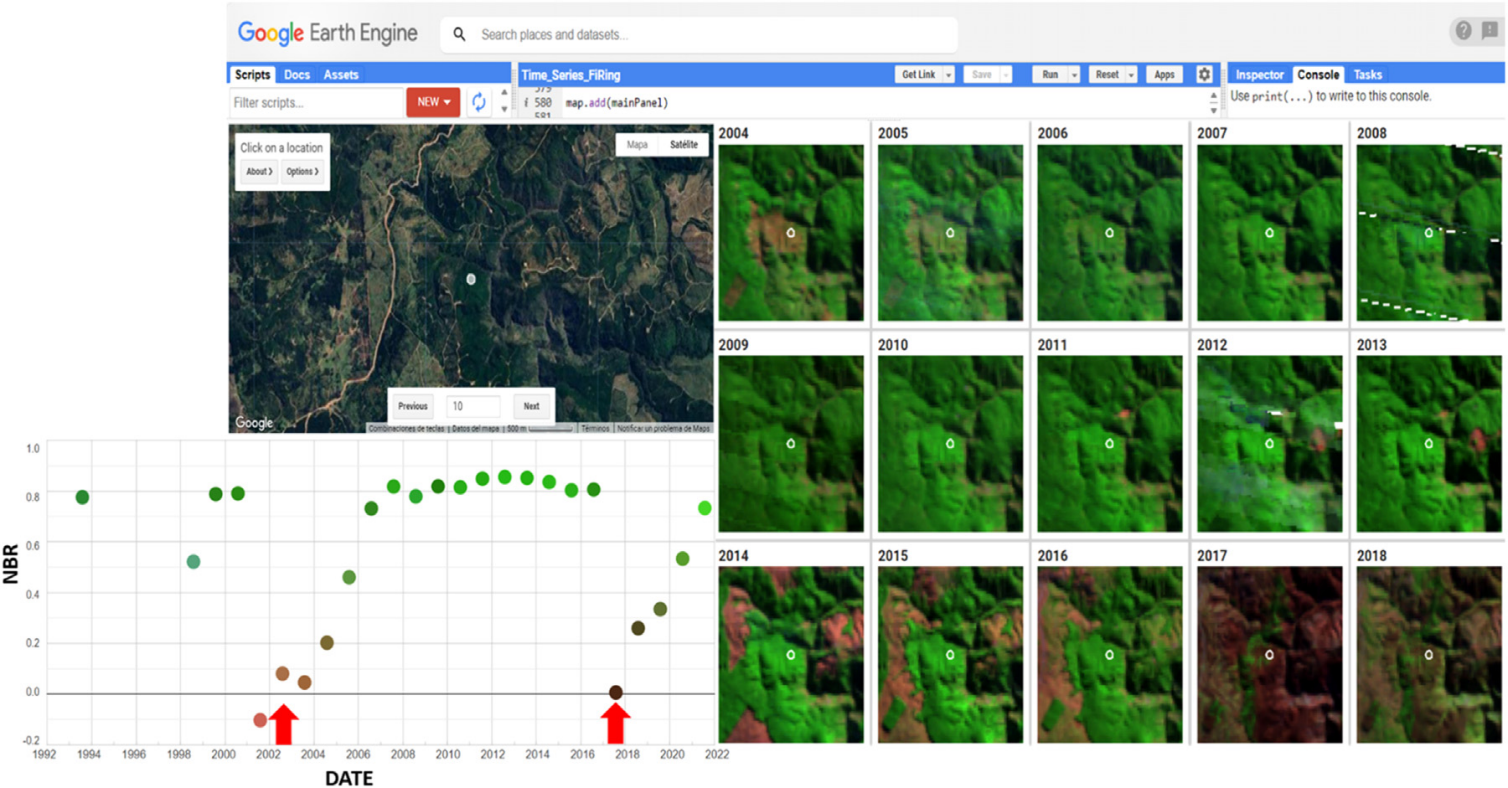
The time series analysis algorithm (LandTrendr) developed by Braaten (2021) is used to monitor and evaluate land use change and burned areas [7,24,43]. The behavior graph obtained indicates Normalize Burn Ratio (NBR) each year of the chronosequence (dots in the graphs). Green dots indicate higher NBR and higher green vegetation cover. Brown dots indicate lower NBR and less green vegetation in the study area. Red arrows point to the years where lower NBR was predicted, indicating the possibility of a fire event occurring in those years.

To avoid assessing wildfire occurring in the same select site (avoid recurrence), the remote sensing LandTrendr analysis [24], was performed, and annual satellite images released a behavior graph of the dNBR indicator (Fig. 2). The algorithm is based on a combination of linear and non-linear regression models and temporal smoothing techniques to detect vegetation changes based on a time-series Landsat image trajectory. The implementation in GEE enables fast and efficient large-scale computation, allowing detailed and accurate assessment of land cover, land use changes, and burned areas over time. The behavioral graph describes NBR, obtained at the same date as the selected severity scar, as an index where a high value is related to a high greenness area with high vegetational cover land, and low NBR values related to brown and black area indicate low vegetation cover and visible soil. The fire event is observed as a sudden decrease in the NBR index. The recovery of trees is identified as a consistent increase in the NBR index over two to three consecutive years. Distortions of the graphs by omission of images or interferences as clouds were evaluated case by case and corroborated in the field (Fig. S1, Supplementary Materials). The discrimination criteria on the behavior NBR graph were 1) fire recurrence in the same site and 2) land use changes. 3) Eliminate repetitive points by selecting different forest patches.

By applying the criteria shown in Table 3, the number of sites to be analyzed by LandTrendr was reduced, and after the LandTrendr analyses, those sites were even fewer. The fire chronosequence analysis yielded no >20 sites of burned native forests and pine plantations per cluster years analyses as a result of the specificity and the quality control performed by the specific criteria of this study. The information obtained after the remote sensing analyses for moderate and high wildfire severity of Chilean native forest allowed us to identify sites near and larger than two hectares of native forest dominated by a mix of temperate deciduous and evergreen *Nothofagus spp*. (locally named Roble-Rauli-Coigue type), per the chosen cluster season in between the chronosequence (Table S1, supplementary materials).

## Method validation

### Soil and vegetation analyses

**Fig. 3:**
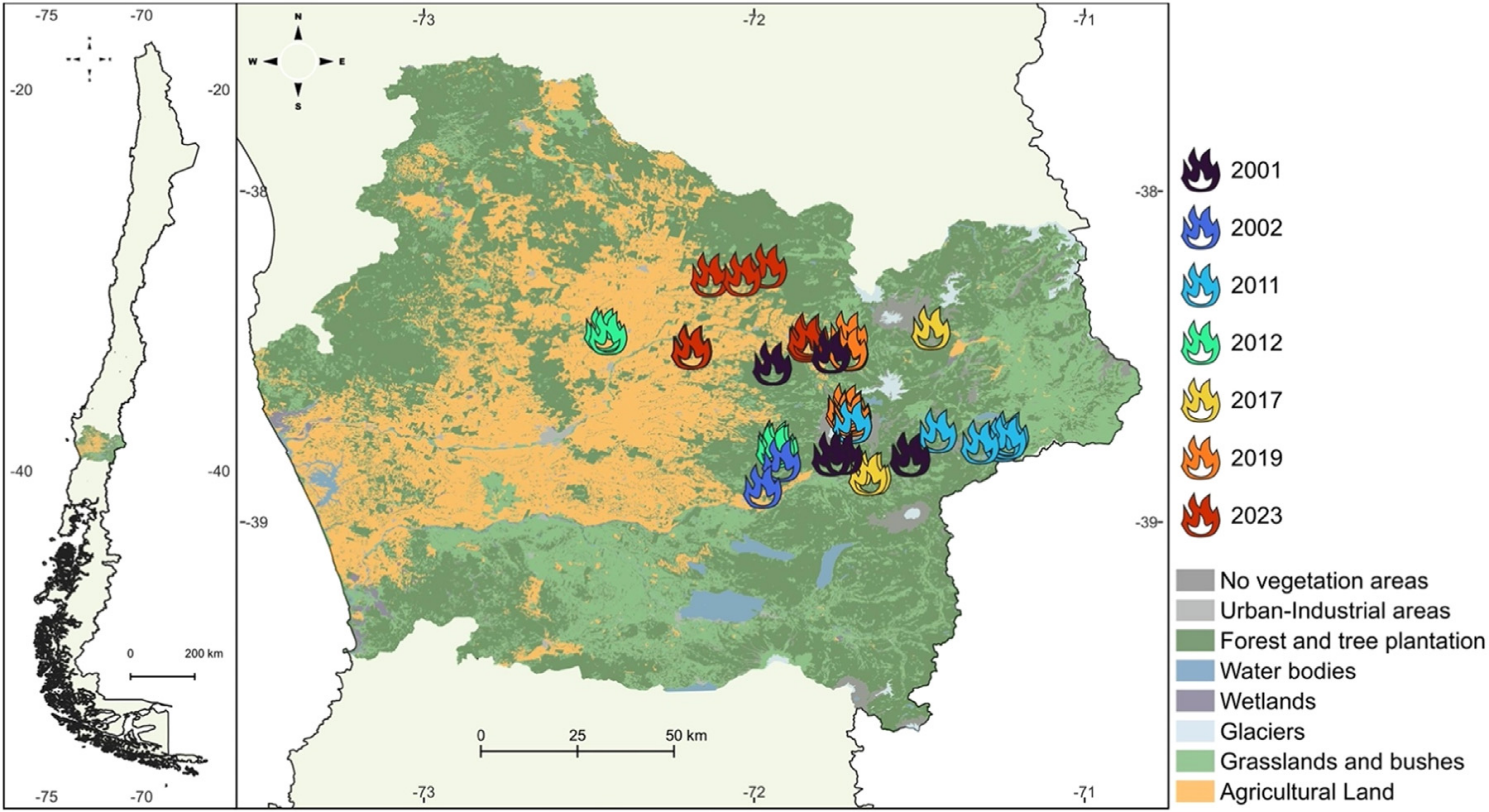
Maps of selected burned native forest sites from the Araucania Region. The land use of forest includes native forest in the survey information. The results of the selective burning sites in native forest land use are presented for the selected years of vegetation and soil sampling under the scope of this study.

To validate the remote sensing selection of sites burned at different times, a selection of 4 sites per cluster season and per condition (burned native forest, burned pine plantation) was selected to perform a field assessment including soil description, classification, sampling, and vegetational inventory for further analyses. Unburned vegetation conditions were selected, evaluated, and sampled in each field to compare affected and unaffected areas (Fig. 3). The Burned and unburned site validation began with a survey of local owners to assess the history of past fire events, magnitude, and postfire or land change management. Field assessment of vegetation cover and soil sampling was conducted between August 2022 and March 2024, from spring to autumn. A total of 40 sites for soil sampling and 20 sites of vegetational monitoring were performed; on these sites, complete soil and vegetation characterization and analyses were described. To evaluate the assertive assessment of wildfire scars on native forests and pine plantations, uppermost soil from the 0–5 cm depth was analyzed on three of the most affected properties after a wildfire [44]. Soil organic carbon on TOC-SSM- 5000A-VCSH (Shimadzu, Kyoto, Japan) by 900 °C soil combustion. In addition, Inorganic C was analyzed by 200 °C soil combustion with hydrochloric acid addition. Bulk density by the cylinder methodology [45]. Finally, dry soils were tested for the wettability test to evaluate the hydrophobicity of soils after a fire [46]. Here, an index on a scale of 1 to 5 was evaluated from wettable soil (1) to extremely hydrophobic soil (5). To evaluate plant production, the depth of the litter layer was measured in situ by placing a plastic ruler till the top horizon of the soil was reached; this was repeated at least 5 times, and the average was calculated. Correlation matrix and lineal model at *p < 0.05* to evaluate those characteristics were performed.

**Fig. 4:**
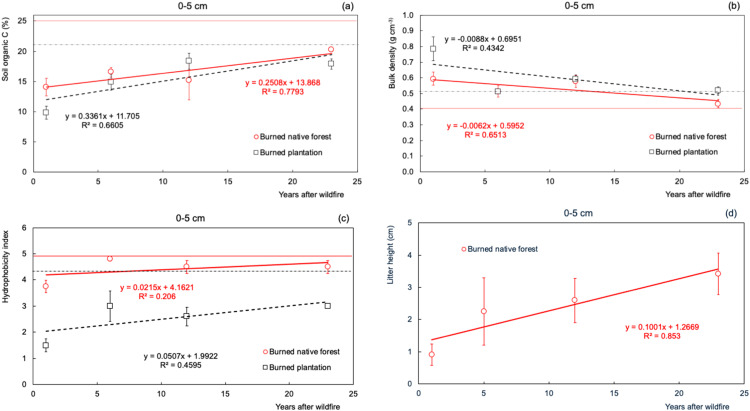
Soil properties were evaluated across a wildfire chronosequence after 23 years of soil and vegetation recovery, as well as soil organic carbon (a) and bulk density (b). Full-draw tendency lines are related to native forests, and segmented tendency lines refer to pine tree plantations. Straight lines represent in red the values of SOC (25 %) and Bd (0.45 g cm^-3^) from the average of unburned native forest sites, and the segmented line represents the values of SOC (21 %) and Bd (0.51 g cm^-3^) from the average of unburned pine plantation. Hydrophobicity index (c) was created on a scale of 1 to 5, from wettable to highly hydrophobic. Litter layer accumulation (d), the average of the litter depth of the burned native forest, is presented on the different cluster seasons.

The *in-situ* soil properties analyses (Fig. 4) validate the selected sites and the chronosequence clusters indicate a consistent recovery of the evaluated soil properties through 23 years. Our results were consistent with the recovery time observed on other studies [[47], [48], [49], [50]].

## Methods limitation

Remote sensing using the proposed computing platform allowed the analysis of large areas for more extended periods than conventional approaches. The burn scars assessment identification was more accurate when the periodicity of satellite images increased after the year 2000, which is why, from 1985 to 1999, only a few years were analyzed.

The field sampling was reduced to 40 sites, 10 sites per season, where four sites of burned native forest, four burned pine plantations, one unburned native forest, and one unburned pine plantation were selected by each season. In addition, eight sites from the season (2005–2006) for vegetation analysis on burned native forests and pine plantations were added to assess regular intervals of vegetation recovery. Possible mistaken areas were related to relief-derived shadows, clouds, wildfire smoke, snow, areas without vegetation and open land, and abrupt land cover conversion related to land use changes or forest harvest. Local knowledge of past events assessed by a field survey allowed for adjusting the criteria to avoid interferences. For instance, from high fire severity to moderate severity observation or when the soil was other than Andisols. However, most selected sites and visited seasons met the selection criteria. In contrast, most of the biases were the smoke shadows due to the high number of wildfires (5 years of recovery) and forest management (e.g. thinning or harvest) (23 years of recovery). *In situ* observation indicates an accurate selection of volcanic soils and forest classification, the main assessment for validation. From the total 40 sites sampled in the chronosequence, we confirmed that 80 % corresponded to the sites selected using the computing Platform and criteria (Table 3). LandTrendr analyses allowed us to understand the forest soil recovery. Other studies, such as [16], rely on the already taken data and its digitalization. Our results lead to assessing the severity of present and past wildfires using remote access locations with the current computing platform in the chronosequence.

Chilean Mediterranean-humid temperate ecosystems (> 38° S) are frequently affected by wildfires due to the expansive conversion of the land use cover matrix and increasing drought season length and severity due to climate change [51]. Even though the study area was located in a high fire-recurrency and susceptible area, we still encountered limitations in our approach. First, previous to the year 2000, few images were available because of a limited number of satellite images between 1985 and 1999, as the periodicity of the first satellite was no more than two pictures a month for this area. The presence of clouds also constrained the availability of images. Second, wildfires are unpredictable, and over time, the severity and affected areas are not homogeneous. To solve those issues, 4 cluster seasons (2023–2021, 2019–2017, 2012–2011, and 2002–2001) were performed, and the soil and vegetational analyses were performed to evaluate the recovery.

## Credit author statement

F. Najera, F. Matus writing and creative processing, E. Duarte, writing, satellite image analyses and suggestion. C. Smith-Ramirez and A. Rendón for supervision and aid during creative processes. P. Pereira, editor and Advisor during the creative and manuscript creation processes. A. Stehr, R. Rubilar, C. Merino, C. Rojas, I. Jofré, F. Aburto for their advice and contributions during the project's development stages and manuscript writing processes.

## Declaration of competing interest

The authors declare that they have no known competing financial interests or personal relationships that could have appeared to influence the work reported in this paper.

## Data Availability

Data will be made available on request.

## Appendix Supplementary materials

Image, application 1

## Footnotes

1 Data set. Duarte E. et al. 2023. Online fire Information report for Chile's fourth region [In Spanish]. https://app.powerbi.com/view?r=eyJrIjoiNzU3ODUyN2ItYzZmYS00MWJjLWJjODItYjk5OGJjY2Q1YjgwIiwidCI6ImFjOGFjOGY5LWFiNDQtNDA3YS1iNThlLTYwY2EzMGVlOGU0YyJ9]
