# Supplementary material for: Multi-temporal assessment of a wildfire chronosequence by remote sensing
Source: MethodsX. 2024 Oct 18;13:103011. doi: 10.1016/j.mex.2024.103011 (PMC11538794; doi:10.1016/j.mex.2024.103011)

Table S1: continuation Remote sensing analyses results from moderate and high wildfire severity of Chilean native forest sites of the Andes range obtained using nine criteria. Native Forests seletion are only shown here.

| **Year** | **Selected severity area (ha)** | **Total area (ha)** | **Forest Structure** | **Surface coverage** | **Type of Forest** | **Long (^o^)** | **Lat (^o^)** | **Elevation (m a.s.l)** | **Exposition (degree)** |
| --- | --- | --- | --- | --- | --- | --- | --- | --- | --- |
| 2001 | 0.83 | 22.77 | Renewal | Dense | Roble-Rauli-Coihue | -71.76506 | -38.82810 | 771 | 180 |
| 2001 | 2.49 | 22.77 | Renewal | Dense | Roble-Rauli-Coihue | -71.76937 | -38.82743 | 761 | 207 |
| 2001 | 1.96 | 22.77 | Renewal | Dense | Roble-Rauli-Coihue | -71.77234 | -38.82959 | 729 | 165 |
| 2001 | 0.61 | 194.85 | Renewal | Dense | Roble-Rauli-Coihue | -71.72219 | -38.88410 | 902 | 2 |
| 2001 | 0.75 | 2.24 | Renewal | Dense | Roble-Rauli-Coihue | -71.91274 | -38.91344 | 391 | 8 |
| 2001 | 1.48 | 180.39 | Adult | Dense | Coihue-Rauli-Tepa | -71.95883 | -38.57067 | 856 | 278 |
| 2001 | 1.03 | 3.71 | Renewal | Semidense | Roble-Rauli-Coihue | -71.96206 | -38.88295 | 562 | 168 |
| 2011 | 1.00 | 2.45 | Renewal | Semidense | Roble-Rauli-Coihue | -71.42927 | -38.82959 | 992 | 199 |
| 2011 | 1.00 | 50.56 | Renewal | Semidense | Roble-Rauli-Coihue | -71.60256 | -38.74389 | 1468 | 47 |
| 2011 | 1.00 | 22.45 | Adult | Dense | Coihue-Rauli-Tepa | -71.87919 | -38.96245 | 1182 | 95 |
| 2011 | 2.00 | 2465.05 | Renewal | Dense | Roble-Rauli-Coihue | -71.52800 | -38.69619 | 1767 | 294 |
| 2011 | 2.00 | 129.49 | Renewal | Semidense | Roble-Rauli-Coihue | -71.90097 | -38.78044 | 1028 | 93 |
| 2011 | 1.00 | 3.99 | Renewal | Dense | Roble-Rauli-Coihue | -71.51093 | -38.84630 | 706 | 180 |
| 2017 | 2.10 | 2698.74 | Renewal | Dense | Roble-Rauli-Coihue | -71.86208 | -38.95383 | 797 | 243 |
| 2017 | 2.49 | 2698.74 | Renewal | Open | Roble-Rauli-Coihue | -71.85831 | -38.93523 | 810 | 139 |
| 2017 | 0.87 | 2698.74 | Renewal | Semidense | Roble-Rauli-Coihue | -71.85453 | -38.93631 | 664 | 149 |
| 2017 | 7.18 | 7.46 | Renewal | Open | Roble-Rauli-Coihue | -71.65592 | -38.49838 | 1124 | 315 |
| 2017 | 9.01 | 37.15 | Renewal | Open | Roble-Rauli-Coihue | -71.64594 | -38.50437 | 1205 | 214 |
| 2017 | 9.08 | 9.08 | Renewal | Open | Roble-Rauli-Coihue | -71.64675 | -38.50835 | 1188 | 0 |
| 2017 | 2.91 | 56.21 | Adult-Renewal | Dense | Coihue-Rauli-Tepa | -71.66512 | -38.50888 | 1281 | 0 |
| 2019 | 0.69 | 1208.44 | Adult | Dense | Coihue-Rauli-Tepa | -71.89792 | -38.76201 | 932 | 214 |
| 2019 | 1.49 | 1208.44 | Renewal | Dense | Roble-Rauli-Coihue | -71.89414 | -38.7624 | 923 | 247 |
| 2019 | 2.18 | 3.45 | Adult | Dense | Coihue-Rauli-Tepa | -71.89846 | -38.51751 | 1047 | 211 |
| 2019 | 1.53 | 1.62 | Adult-Renewal | Dense | Coihue-Rauli-Tepa | -72.13911 | -38.60806 | 384 | 0 |
| 2019 | 1.93 | 335.09 | Adult | Dense | Coihue-Rauli-Tepa | -71.90951 | -38.56467 | 1095 | 225 |
| 2021 | 0.64 | 6.87 | Renewal | Open | Roble-Rauli-Coihue | -71.76668 | -38.8083 | 1114 | 90 |
| 2021 | 0.77 | 38.37 | Adult-Renewal | Open | Coihue-Rauli-Tepa | -71.86887 | -38.74645 | 955 | 183 |
| 2021 | 0.87 | 38.37 | Adult-Renewal | Dense | Coihue-Rauli-Tepa | -71.86397 | -38.74928 | 985 | 180 |
| 2021 | 6.34 | 20.48 | Renewal | Open | Roble-Rauli-Coihue | -71.6039 | -38.48733 | 1210 | 45 |
| 2021 | 3.95 | 22.09 | Stubby | Dense | Lenga | -71.86154 | -38.53665 | 1325 | 0 |
| 2021 | 2.42 | 22.09 | Stubby | Dense | Lenga | -71.86262 | -38.53099 | 1266 | 315 |

Table S1 continuation: Aditional information obtained after the remote sensing analyses for moderate and high wildfire severity of Chilean native forest sites of the Andes range derived of using various criteria (Table 3 in the text) To each location, the closest pine tree plantations were also sampled (data not shown).

| **Year of the fire** | **Fire (ha)** | **Severity class** | **Canopy Heght (m)** | **Type of Forest** | **Subtype of forest** | **Dominan Species** | **Nerest town** | **Highway (km)** |
| --- | --- | --- | --- | --- | --- | --- | --- | --- |
| 2001 | 0.83 | 5 | 04--08 | Roble-Rauli-Coihue | Roble-Rauli-Coihue | Nothofagus dombeyi | Melipeuco | 2.4 |
| 2001 | 2.49 | 5 | 12--20 | Roble-Rauli-Coihue | Roble | Nothofagus obliqua | Melipeuco | 2.4 |
| 2001 | 1.96 | 5 | 12--20 | Roble-Rauli-Coihue | Roble | Nothofagus obliqua | Melipeuco | 2.2 |
| 2001 | 0.61 | 5 | 12--20 | Roble-Rauli-Coihue | Roble | Nothofagus obliqua | Melipeuco | 1.5 |
| 2001 | 0.75 | 5 | 04--08 | Roble-Rauli-Coihue | Roble | Nothofagus obliqua | Cunco | 0.1 |
| 2001 | 1.48 | 6 | 20 - 32 | Coihue-Rauli-Tepa | Coihue-Rauli-Tepa | Nothofagus dombeyi | Lautaro | 1.4 |
| 2001 | 1.03 | 6 | 08--12 | Roble-Rauli-Coihue | Coihue | Nothofagus dombeyi | Cunco | 1.1 |
| 2011 | 1.00 | 5 | 08--12 | Roble-Rauli-Coihue | Coihue | Nothofagus dombeyi | Melipeuco | 0.2 |
| 2011 | 1.00 | 5 | 12--20 | Roble-Rauli-Coihue | Roble-Rauli-Coihue | Nothofagus alpina | Melipeuco | 1.8 |
| 2011 | 1.00 | 5 | 20 - 32 | Coihue-Rauli-Tepa | Coihue-Tepa | Nothofagus dombeyi | Cunco | 2.1 |
| 2011 | 2.00 | 5 | 12--20 | Roble-Rauli-Coihue | Roble-Rauli-Coihue | Nothofagus alpina | Melipeuco | 2.6 |
| 2011 | 2.00 | 5 | 08--12 | Roble-Rauli-Coihue | Coihue | Nothofagus dombeyi | Vilcun | 0.1 |
| 2011 | 1.00 | 5 | 12--20 | Roble-Rauli-Coihue | Roble-Rauli-Coihue | Nothofagus dombeyi | Melipeuco | 0.4 |
| 2017 | 2.10 | 5 | 04--08 | Roble-Rauli-Coihue | Coihue | Nothofagus dombeyi | Cunco | 0.9 |
| 2017 | 2.49 | 5 | 04--08 | Roble-Rauli-Coihue | Roble | Nothofagus obliqua | Cunco | 0.8 |
| 2017 | 0.87 | 5 | 08--12 | Roble-Rauli-Coihue | Coihue | Nothofagus dombeyi | Cunco | 0.6 |
| 2017 | 7.18 | 5 | 12--20 | Roble-Rauli-Coihue | Roble-Rauli-Coihue | Nothofagus alpina | Curacautin | 0.1 |
| 2017 | 9.01 | 5 | 12--20 | Roble-Rauli-Coihue | Roble-Rauli-Coihue | Nothofagus alpina | Curacautin | 0.0 |
| 2017 | 9.08 | 5 | 12--20 | Roble-Rauli-Coihue | Roble-Rauli-Coihue | Nothofagus alpina | Curacautin | 0.0 |
| 2017 | 2.91 | 5 | 12--20 | Coihue-Rauli-Tepa | Coihue-Rauli-Tepa | Nothofagus dombeyi | Curacautin | 1.2 |
| 2019 | 0.69 | 5 | 20 - 32 | Coihue-Rauli-Tepa | Coihue | Nothofagus dombeyi | Vilcun | 0.1 |
| 2019 | 1.49 | 5 | 08--12 | Roble-Rauli-Coihue | Coihue | Nothofagus dombeyi | Vilcun | 0.2 |
| 2019 | 2.18 | 5 | 20 - 32 | Coihue-Rauli-Tepa | Coihue-Rauli-Tepa | Nothofagus dombeyi | Curacautin | 3.1 |
| 2019 | 1.53 | 5 | 12--20 | Coihue-Rauli-Tepa | Coihue-Rauli-Tepa | Nothofagus obliqua | Vilcun | 0.9 |
| 2019 | 1.93 | 5 | 20 - 32 | Coihue-Rauli-Tepa | Coihue-Rauli-Tepa | Nothofagus dombeyi | Lautaro | 6.1 |
| 2021 | 0.64 | 5 | 02--04 | Roble-Rauli-Coihue | Coihue | Nothofagus dombeyi | Melipeuco | 4.4 |
| 2021 | 0.77 | 7 | 12--20 | Coihue-Rauli-Tepa | Coihue | Nothofagus dombeyi | Vilcun | 3.1 |
| 2021 | 0.87 | 6 | 12--20 | Coihue-Rauli-Tepa | Coihue | Nothofagus dombeyi | Vilcun | 3.2 |
| 2021 | 6.34 | 5 | 02--04 | Roble-Rauli-Coihue | Roble-Rauli-Coihue | Nothofagus dombeyi | Curacautin | 1.0 |
| 2021 | 3.95 | 5 | 02--04 | Lenga | Ñirre | Nothofagus antarctica | Curacautin | 2.2 |
| 2021 | 2.42 | 5 | 02--04 | Lenga | Ñirre | Nothofagus antarctica | Curacautin | 2.4 |

Map S1 Maps of soil series classified with USDA soil Taxonomy system obtaned by local geoinformation.


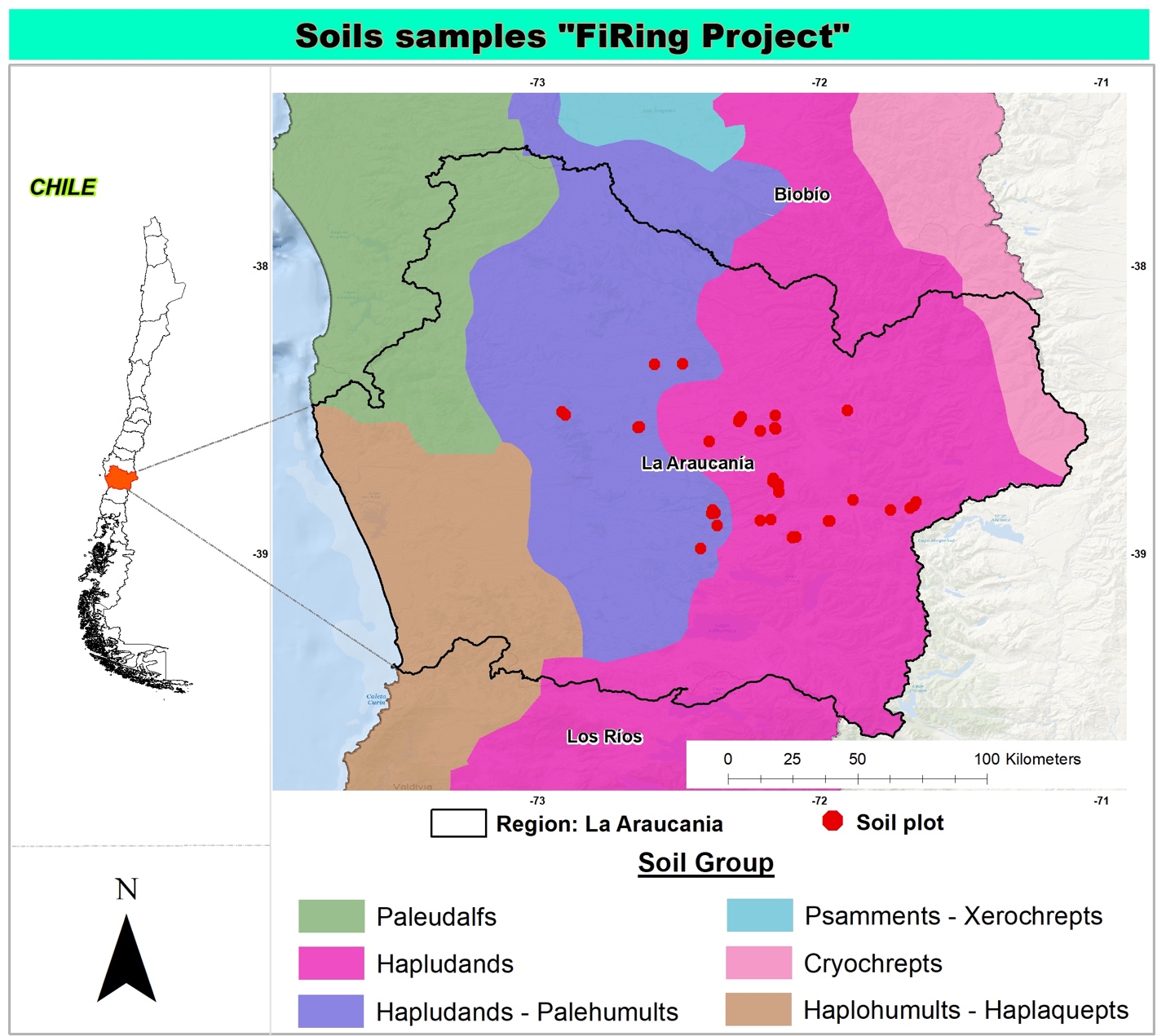


Map S2 Map of slope of the Araucania region obtained with a Digital Elevation Model (DEM) from a geographical information of contour lines separated at 50 m. The calculation were performed on QGIS software.


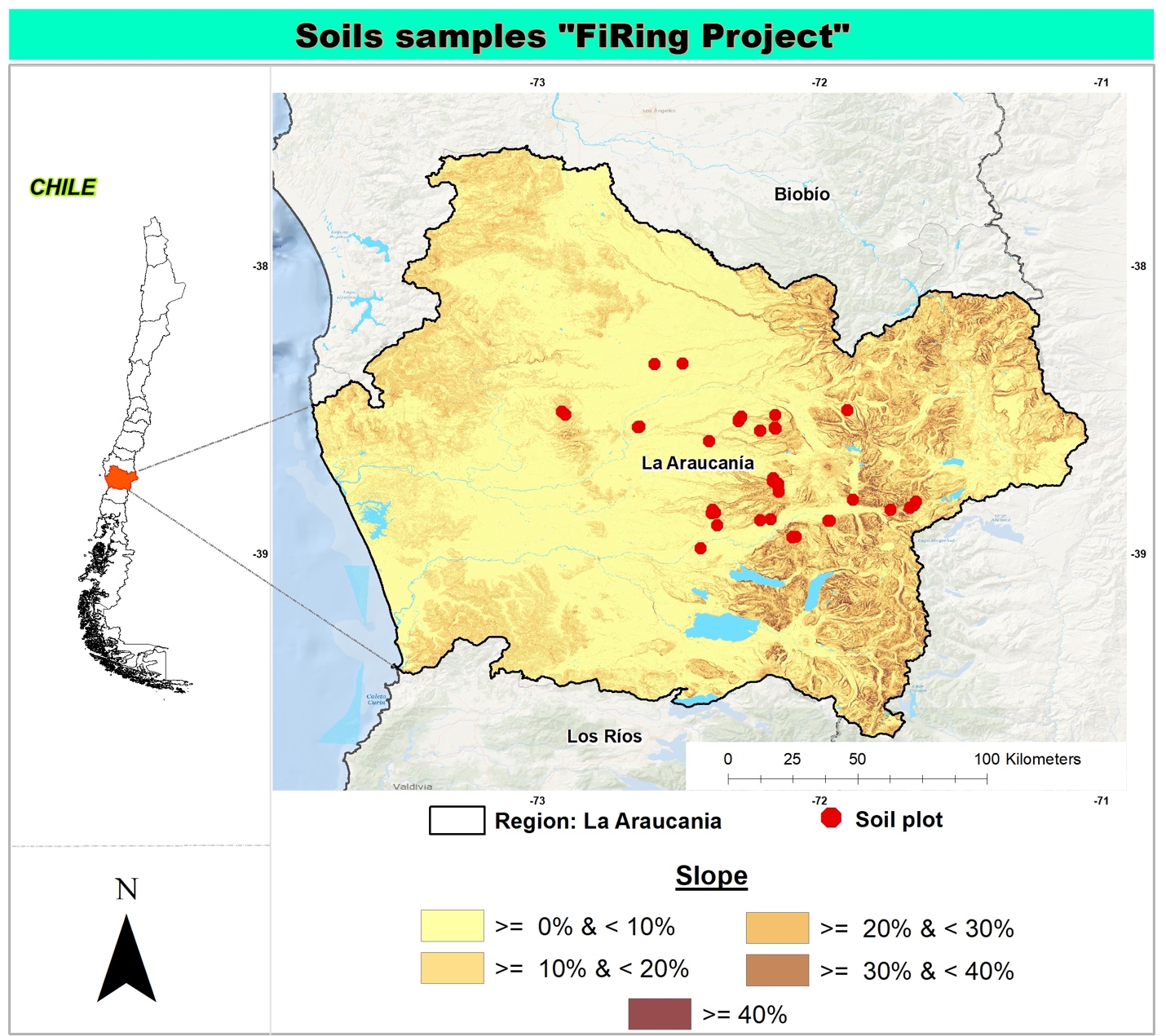


Map S3 of aspect of the Araucania region obtained with a Digital Elevation Model (DEM) from a geographical information of contour lines separated at 50 m. The calculation were performed on QGIS software.


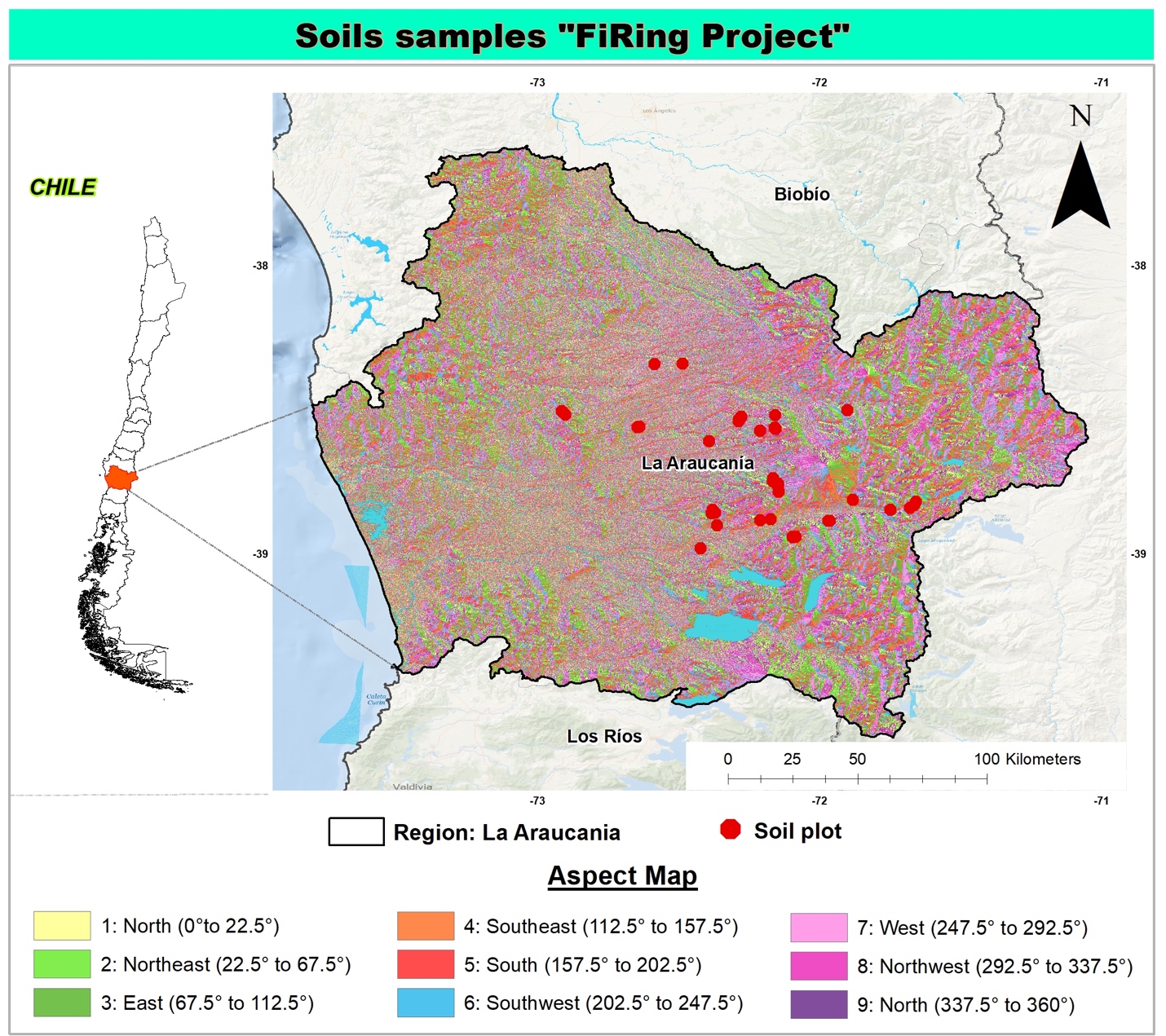


Figure S1: Correlation matrix of soil properties and ecosystem recoverty time after a wildfire under the selective criterias of the scope of this study. *indicate significative correlation.

**Pearson's r heatmap**


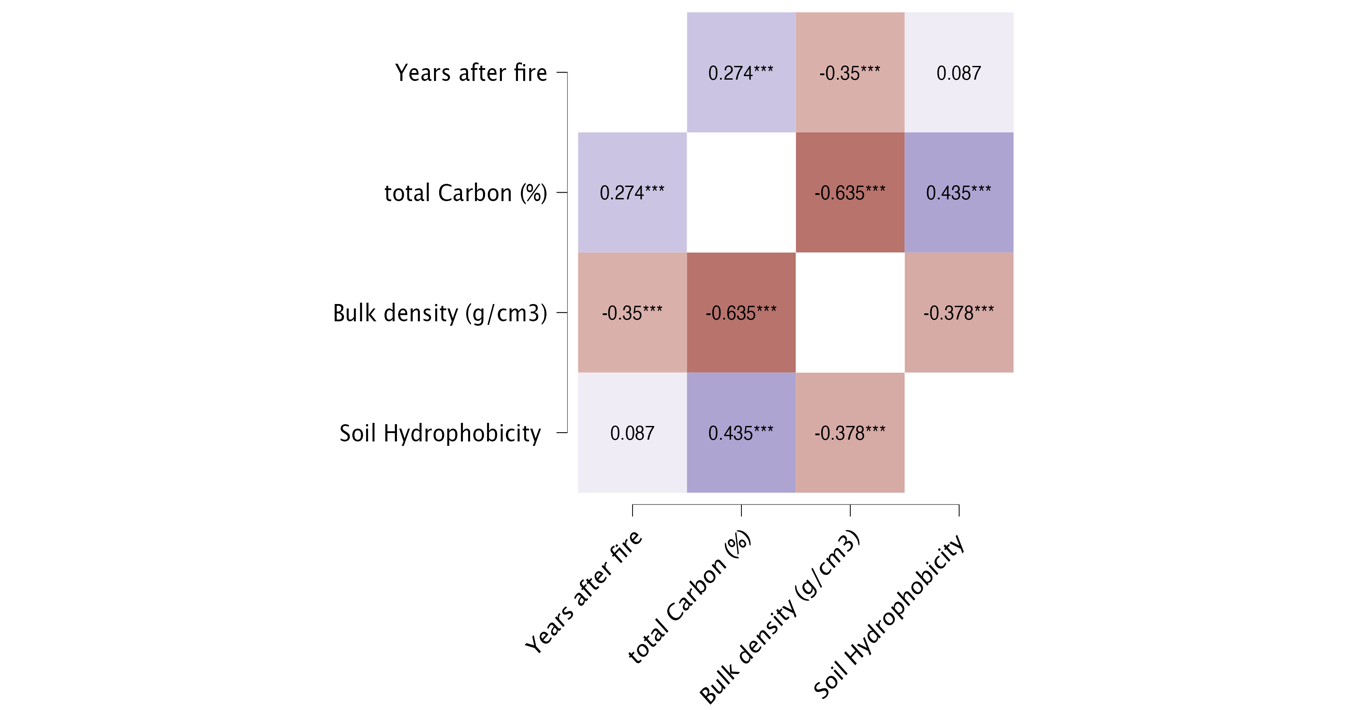

Supplement: Supplementary file 1 [file mmc1.docx]
